# Influence of Implant Thread Morphology on Primary Stability: A Prospective Clinical Study

**DOI:** 10.1155/2020/6974050

**Published:** 2020-08-05

**Authors:** Maria Menini, Francesco Bagnasco, Ivan Calimodio, Nicolò Di Tullio, Francesca Delucchi, Domenico Baldi, Francesco Pera

**Affiliations:** ^1^Department of Surgical Sciences (DISC), Implant and Prosthetic Dentistry Unit, University of Genoa, Ospedale S. Martino (pad. 4), L. Rosanna Benzi 10, 16132 Genoa, Italy; ^2^Interdepartmental Research Center, Dental-School, University of Turin, Turin, Italy

## Abstract

**Objectives:**

The purpose of this study was to evaluate the primary stability of two implants with the same macro- and micromorphology but different thread design and analyze their clinical outcomes over a one-year period.

**Materials and Methods:**

14 patients needing a partial rehabilitation with a delayed loading approach (DEL group: 9 patients) or a full-arch rehabilitation treated with immediately loaded fixed prostheses supported by 4 implants following the Columbus Bridge Protocol (CBP) (IL group: 5 patients) were included. In each patient, at least one SY (implant with standard threads) and one SL implant (implant with an augmented depth of the threads) were randomly inserted. Primary outcome measures were the number of threads exposed at a torque of 30 Ncm and 50 Ncm and final insertion torque. Secondary outcome measures were implant and prosthetic failure, peri-implant bone resorption, and periodontal parameters: bleeding on probing (BoP), plaque index (PI), and probing depth (PD) evaluated at 3, 6, and 12 months of healing.

**Results:**

Nineteen SY and 19 SL implants were inserted in 14 patients. Twenty implants (10 SL and 10 SY) were inserted in the IL group, while 18 (9 SL and 9 SY) were inserted in the DEL group and followed-up for 12 months. No patients dropped out. No implants and prostheses failed. No biological complications were identified. No significant differences were found between SY and SL implants comparing the number of exposed threads when inserting the implant with a torque insertion of 30 N (*T* student test *p* = .142 and *U* test *p* = .164). At 50 N, no threads were visible in either groups. Final torque insertion values were higher for SL (mean: 48.42 Ncm) compared to SY implants (mean: 43.42 Ncm) without a statistically significant difference. All the implants showed good clinical outcomes at the 1-year-in-function visit.

**Conclusions:**

After 12 months of function, both implant types provided good clinical outcomes without statistically significant differences between the two groups. A difference in insertion torque (even if not statistically significant) was found with higher insertion torque values for SL implants with a larger thread depth.

## 1. Introduction

Dental implant rehabilitation is considered a highly predictable method to replace missing teeth, with a success rate ranging around 95% [1]. Adequate implant stability with a close connection between residual peri-implant bone and the implant itself, avoiding micromovements of the implants, is a fundamental prerequisite to achieve successful osseointegration [2, 3].

Primary implant stability, that is, the mechanical stability of the implant at placement, is a mechanical phenomenon that can be determined by measuring insertion torque values [4]. It mainly depends on three factors [5]:

1. biomechanical properties of bone (quality and quantity of the receiving bone)
2. preparation technique of the implant site (diameter of the largest drill used, length of the preparation, morphology of the drill, and tapping or not of the implant site)
3. macrostructure of the implant (diameter, length, and shape)

While bone characteristics are not modifiable, the surgical technique and the macrostructure of the implant (that is, the macroscopic shape of the implant, including diameter, length, shape, and thread design) and the microstructure of the implant (that is, implant surface characteristics) can be modulated by the clinician to optimize primary stability. Moreover, after implantation, and even once osseointegration has been reached, bone undergoes a constant remodeling which is also influenced by occlusal loads, abutment characteristics, platform switching, etc. [5–9].

Reaching optimal primary stability is a prerequisite particularly important in full-arch immediate loading rehabilitations. In this case, primary stability is considered a key factor together with the use of a rigid framework in order to avoid micromotions of the implants and achieve osseointegration [10–13].

Implant design is one of the key factors to modulate primary stability and stress distribution to peri-implant bone. The geometric features of an implant strongly affect its surface area, and as a consequence, they influence the amount of bone-implant contact (BIC). Implants with deeper threads, small pitch, and reduced helix angle were shown to enhance primary stability by achieving higher bone to implant contact while reducing osseocompression [14, 15].

Implant geometry also plays an important role on stress distribution at the bone-implant interface and on implant capacity to withstand forces during the process of osseointegration. Therefore, implant thread design affects both the obtainment and maintenance of osseointegration through multiple mechanisms [5, 14].

Clinically, implant stability can be measured with torque insertion force or resonance frequency analysis. Insertion torque values are very important for the clinical determination of primary stability levels and the absence of micromovement whenever an immediate load is applied [16].

The purpose of the present study was to evaluate the primary stability of two implants with the same micro- and macromorphology but different thread designs and investigate the relation of implant thread design with clinical outcomes over a one-year period. The null hypothesis tested was that there was a difference neither in torque insertion nor in 1-year clinical outcomes between dental implants with standard and increased depth of the threads.

## 2. Materials and Methods

In the period between May 2017 and March 2019, patients referring to the Implant and Prosthetic Dentistry Unit of the Department of Surgical Sciences (DISC) of Genoa University were selected if they required the insertion of at least two implants. This prospective study was performed following the principles outlined in the Declaration of Helsinki on experimentation involving human subjects. All patients were thoroughly informed about the procedures and signed an informed consent form.

Exclusion criteria were as follows:

- patients with a history of bisphosphonate therapy
- patients with uncontrolled diabetes (HbA1c > 6%, glycemic level > 110 mg/dL)
- patients with relevant medical conditions contraindicating oral surgery
- patients without sufficient native bone needing regenerative procedures

### 2.1. Implant Characteristics

**Figure 1:**
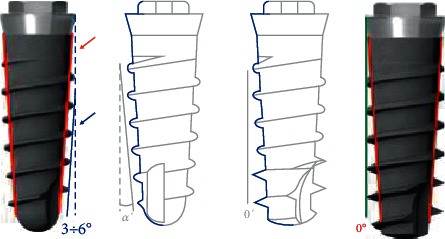
Design of the two tested implants: SY on the left and SL on the right.

The titanium implants analyzed (Syra and Syra SL, Sweden & Martina, Due Carrare PD, Italy) presented an external hexagon connection, a conical morphology, and a surface sand-blasted with zirconia oxide and etched with mineral acid (Figure 1). The neck was machined for the height of 1.00 mm and presented a divergent shape with different angles according to the implant diameter, in order to use the same prosthetic component on all implant diameters:

- Implant diameter of 3.80 mm and platform diameter of 4.10 mm ➔ collar divergence of 14°
- Implant diameter of 4.25 mm and platform diameter of 4.10 mm ➔ collar divergence of 7.5°
- Implant diameter of 5.00 mm and platform diameter of 5.00 mm ➔ collar divergence of 7.5°

The SYRA implant (SY) had a constant thread depth of 0.25 mm along the whole body of the fixture, keeping the maximum external profile of the implant conical.

Syra SL (SL) implants differ from SY for two factors:

- Depth of the threads: it gradually increases from 0.25 mm in the coronal part of the implant body to 0.70 mm in the apical part, making the maximum profile of the implant cylindrical
- Shape of the threads: trapezoidal in the loops in the upper part of the implant (such as SY implants) and triangular shape in the apical part. On the contrary, SY implants present a trapezoidal shape of the threads throughout their entire length

Patients were divided into two groups depending on the rehabilitation required:

- DEL group: patients needing a partial rehabilitation with a delayed loading approach
- IL group: patients needing a full-arch rehabilitation treated with immediate loading full-arch fixed prostheses supported by implants (*n* = 4‐6 for an arch) following the Columbus Bridge Protocol (CBP) [10, 13]

**Figure 2:**
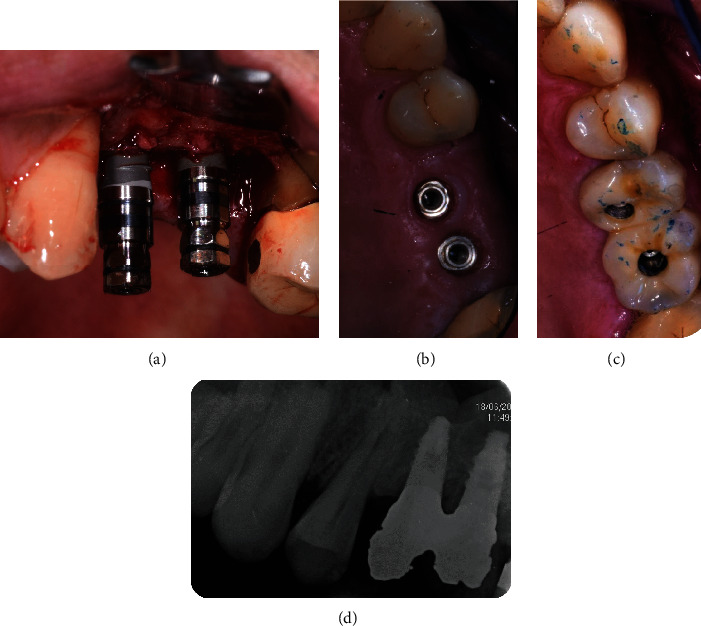
Clinical pictures of one of the patients included in the present research (DEL group): (a) clinical image of insertion of the 2 implants at 30 Ncm (T0); (b) healing phase (3 months after implant insertion); (c) delivery of the fixed prosthesis (14 weeks after implant insertion); (d) radiographic image (1 year after implant insertion).

In each patient, at least one SY and one SL implant were randomly inserted applying a split-mouth methodology (Figure 2). In the IL group, one hemiarch was treated with 2 SY implants and the contralateral hemiarch was treated with 2 SL implants. Each hemiarch was randomly allocated to the SY or SL treatment. In the DEL group, one SL implant and one SY implant were inserted one next to the other into an edentulous area, and their position in the adjacent osteotomies was randomly allocated. Implant length ranged between 10 and 18 mm (10-13 mm in the DEL group and 10-18 mm in the IL group), and implant diameter was 3.8 mm or 4.25 mm depending on available bone.

Under local anesthesia, a crestal incision and a full-thickness flap elevation were performed. The implant sites were prepared starting with a pilot drill followed by the sequence of burs provided by the implant manufacturer. Bone quality was evaluated, and the site preparation protocol was chosen accordingly. Bone was considered according to the Misch [17] classification which is based on the microstructural characteristics of the two components of bone (cortical and spongy bone) and dividing bone quality into 4 types: D1 (dense cortical bone and poor spongy bone), D2 (thick spongy and narrow-meshed cortical bone), D3 (thin-meshed spongy cancellous bone), and D4 (loose cancellous bone). Implants were inserted using the Implantmed electronic surgical drilling unit with torque control (Implantmed, W&H). The device was first set at 30 Ncm, and the implant insertion procedure was stopped when the 30 Ncm insertion torque was reached in order to record the number of exposed threads at 30 Ncm; that is to say, the number of threads above the bone crest was recorded. The same procedure was conducted at 50 Ncm. If the implant was still not in its final position, a manual insertion device was then used and the final insertion torque was registered. The implant threads were observed on the vestibular side of each implant.

### 2.2. DEL Rehabilitation

In the DEL group, a one-stage technique was applied. After implant insertion, transmucosal titanium healing abutments were immediately connected to the implants and soft tissues were approximated and sutured around them. Patients were prescribed analgesics and antibiotic coverage (amoxicillin 2 g/daily or in case of allergy clindamycin 600 mg/daily) for 7 days from the day before surgery, as well as oral rinses of 0.12% chlorhexidine gluconate for 7 days from the day following implant placement. Three months after surgery, a traditional impression was taken, and definitive screw-retained prostheses provided with a metal framework and composite resin veneering material were delivered. All the restorations were splinted; no single crowns were realized.

### 2.3. IL Rehabilitation

In the case of full-arch immediate loading rehabilitations, the Columbus Bridge Protocol was applied [10–13].

The CBP is a surgical and prosthodontic protocol developed for rehabilitation of atrophic, edentulous maxillae, and mandibles using distal tilted implants (upper jaw: implants placed parallel to the anterior sinus wall; lower jaw: implants placed obliquely angled above the mental foramen). The surgical and prosthetic protocol was the same used in already published papers [10–13].

Conical abutments with a 0, 15, or 30 degrees inclination (P.A.D, Sweden & Martina, Due Carrare, Padova) were placed onto the implants immediately after implant insertion, prior to suturing the mucoperiosteal flaps, and a pick-up impression was taken using impression plaster [18]. The fixed screw-retained prostheses delivered 24 hours after surgery were fabricated with a rigid metal framework in order to provide increased strength and rigidity to the prostheses and a composite resin veneering material [2].

The prostheses did not present distal cantilevered extensions.

The drug therapy prescribed was the same as in the delayed loading group. After implant placement, all patients received oral and written recommendations to improve healing: liquid/soft diet for 40 days and hygienic instruments and techniques appropriate to the stage of healing [19].

### 2.4. Outcomes

Primary outcome measures were as follows:

- number of threads exposed at a torque of 30 and 50 Ncm
- final insertion torque

Secondary outcome measures were as follows:

- implant and prosthetic failure
- peri-implant bone resorption calculated using intraoral digital periapical radiographs at the following time points: at implant insertion (T0), at 3, 6, and 12 months of healing. Radiographs were obtained with a parallel long-cone technique. The implant-abutment interface was used as the reference point for bone level measurements. Interproximal bone levels were assessed from these reference points to the most coronal bone levels at the mesial and distal surfaces of each implant. Digital software (OrisWin DG, FONA-Dental, Assago, Italy) was used to perform measurements. The software was calibrated for every image using the implant diameter as a reference. Two of the authors (FB and IC) performed all MBL measurements on the mesial and distal surfaces of each implant after a calibration exercise demonstrating 95.9% concordance within ±0.5 mm for measurements. The examiners were not blinded because the different implant thread morphologies were visible on the radiograph; measurement differences were discussed among examiners until an agreement was found
- periodontal parameters: bleeding on probing (BoP), suppuration, plaque index (PI), and probing depth (PD) evaluated at 3, 6, and 12 months of healing. BoP was defined as the presence of bleeding (yes/no) evaluated at four points for each implant (mesial, distal, buccal, and lingual) using a nonmetallic probe. PI was defined as the presence of plaque (yes/no) on four points using an erythrosine gel. Therefore, for PI and BoP, values from 0 to 4 were recorded for each implant site. PD was assessed at four points for each implant

### 2.5. Statistical Methods

The descriptive statistical analysis included age, gender, loading type, implant position, implant length, bone quality, and implant type (SY or SL). Moreover, peri-implant health parameters such as BOP, PD, PI, suppuration, and bone resorption were analyzed. The main outcomes of the study, i.e., the insertion strength (expressed in Ncm) and the implant exposition at a torque of 30 and 50 Ncm (expressed in a number of threads), were considered. The nonparametric Mann-Whitney test was performed to analyze all evaluated criteria among the groups at each time point. The Kruskal-Wallis test was applied too. ANOVA was used to assess intergroup variability. Linear mixed models were used to investigate differences over time. A significance level of 5% was adopted in all tests, and SPSS IBM (version 25) was used.

## 3. Results

Fourteen patients (8 males and 6 females, mean age: 61.7 years) fulfilled the inclusion criteria and were enrolled in the present research. All the patients attended the follow-up appointments and were followed-up for at least 12 months.

Five patients were rehabilitated in the IL group, while 9 in the DEL group. Baseline characteristics are reported in Table 1.

Twenty implants (10 SL and 10 SY) were inserted in the IL group, while 18 (9 SL and 9 SY) were inserted in the DEL group. In the IL group, two patients rehabilitated the inferior arch and three the upper arch. In the DEL group, 5 patients rehabilitated the upper arch and 4 the lower arch.

The chi-square approximation of the Kruskal-Wallis test did not find significant differences in torque insertion between bone sites with different bone qualities (*p* = .559). An analysis between classes was carried on, and no value was statistically different (Table 2).

Torque insertion data are reported in Table 3. Parametric and nonparametric tests showed a not significant difference between SYRA and SYRA SL in the number of visible threads when inserting the implant with an insertion torque of 30 Ncm (*T* student test: *p* = .142; *U* test: *p* = .164). At 50 Ncm, all the implants had reached their final position in the implant site and no threads were visible in either of the groups.

Parametric and nonparametric tests showed a relevant but not significant difference between SYRA and SYRA SL in final torque insertion values (*T* student test: *p* = .055; *U* test: *p* = .063).

No prosthetic nor implant failures occurred during the follow-up period, and no technical nor biological complications were encountered. Mean peri-implant health parameters are reported in Table 4.

As a consequence, it can be stated that the null hypothesis has not been rejected. In fact, no significant differences were found between SL and SY implants.

**Figure 3:**
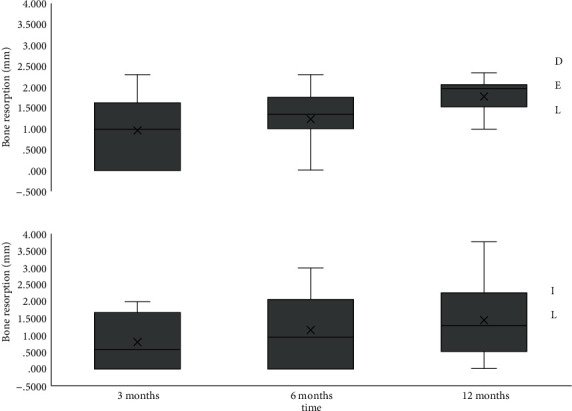
Bone resorption trend over time grouping for DEL and IL rehabilitations. Mean value (X), median value (line in the box), inter 2nd and 3rd quartile range (box), and max and min values (whiskers).

Linear mixed model (interaction time∗loading protocol) was used to investigate the difference in bone resorption over time between DEL and IL. The relationship was significantly different (*p* = .040). DEL showed higher values of bone resorption over time compared to IL (Figure 3).

## 4. Discussion

The results of the present research identified a difference, even if not statistically significant, of torque insertion values between the two implant morphologies. Applying an insertion torque of 30 Ncm, SL implants presented a greater number of exposed threads compared to SY implants, and the final insertion torque was higher for SL implants (48.42 vs. 43.42 Ncm). This indicates that with the same bone quality and the same bone site preparation, implant insertion is easier when using a reduced thread depth while implants with a larger thread diameter need higher insertion torque values and thus reach greater primary stability. This might be due to their increased surface area.

Periodontal parameters were similar for the two implants, and no cases of peri-implantitis or mucositis were noted [20, 21]; no differences in bone resorption were found among the two implants; however, it is interesting to note a significant correlation between bone resorption and loading protocol, with greater bone resorption in the DEL group. At 12 months of postimplant insertion, our analysis reported mean bone resorption of 1.909 mm in the DEL group and 1.440 mm in the IL group. However, it must be considered that only 5 patients were included in the IL group. Moreover, loading time was not the unique difference between DEL and IL groups. The two groups also differed in implant length (longer implants in the immediate loading rehabilitation) and in the type of rehabilitation (partial vs. full-arch prostheses). In addition, in the DEL group, bone resorption around one of the implants might have influenced bone level next to the adjacent implant.

The SY and SL implants used in the present research have a conical shape and, as concerns SL implant, an aggressive design of the threads: this macrostructure has the aim of improving primary stability. The divergent shape of SY and SL implant collar has the purpose of further increasing primary stability while impacting cortical bone. This is in contrast with modern implants with convergent collars aimed at providing greater space for peri-implant tissue in the transmucosal area, in order to favour soft tissue thickness and aesthetic [22].

Primary stability is one of the main factors to be sought in implant insertion in order to favour osseointegration in both single and full-arch rehabilitations. An ideal implant design should provide a balance between compressive and tensile forces while minimizing shear force generation [23, 24].

As reported by McCullough and Klokkevold, implant macrogeometry plays a fundamental role: variations in implant length, diameter, number of threads, thread depth, pitch, and helix angle may strongly influence primary stability [25]. The threads of the implants seem to have a huge relevance in the obtainment of implant osteointegration improving initial stability, maximizing BIC, and favouring stress distribution at the bone-implant interface.

In a FEM study, Huang et al. showed that implant thread morphology is important both during the insertion of the implant to allow its sliding into the implant site and to reduce the stress of peri-implant bone, increasing implant stability and long-term survival [26].

Similar results were provided by Lee et al. [15] showing that implants with greater thread depth provide higher primary stability, especially in low-quality bone. The use of implants with a greater depth of the threads seems also to induce an increased condensation of peri-implant bone. This is in accordance with the results of the present research reporting a greater insertion torque when using SL implants.

In a study by Makary et al., it is reported that the use of large-thread implants turns out to be an advantage only in implant rehabilitations with bone type D3 and D4, while in D1, it appeared to be a disadvantage because of the necessity of an excessive surgical preparation to which bone should be subjected [27].

However, it is difficult to understand what the ideal depth of the threads should be in function of bone density to ensure high primary stability and a better distribution of the stresses into the peri-implant bone.

Ao et al. in a FEM analysis evaluated the behaviour of implants with a depth of threads ranging from 0.2 to 0.6 mm. The study showed that larger threads tend to have a better distribution of peri-implant stress than the narrower ones. The threads with a depth greater than 0.44 mm showed a better biomechanical behaviour, reaching the best results with a depth between 0.34 and 0.5 mm [28].

It is important to note that not only the depth but also the shape of the threads can influence the primary stability of the implant and peri-implant bone resorption. Several studies have shown that immediately after implant placement, occlusal loads are mainly concentrated at the bone next to the first thread, indicating that the implant width and the wires can create a reduction of loads [14, 15].

Some studies have analyzed how a different morphology of the threads can determine a different distribution of the loads into peri-implant bone. A more squared shape of the threads allows to increase the BIC and to dissipate loads on a greater bone surface, allowing a better distribution especially of the lateral forces. A V-shape of the threads in the most apical part of the implant determines a greater aggressiveness of the implant, especially in a poor-quality bone, managing to obtain greater mechanical stability of the implant and greater resistance to vertical forces [14, 20, 29]. It must be noted that in the present study, SY and SL implants did not differ for thread depth only. In fact, SY implants present a trapezoidal thread shape throughout their entire length, while SL implant threads show a trapezoidal shape in the coronal portion and a more aggressive triangular shape in the apical part.

Our research failed to find a difference in bone resorption between SY and SL implants. However, SL implants presented greater primary stability, and this may help in reaching good stability in poor quality bone. This is particularly important in immediate loading rehabilitations.

In the present research, the drilling protocol varied according to bone quality as proposed by the manufacturer's guidelines, and the same identical drilling procedure was applied for SL and SY implants in the same patient. This was done in order to reduce possible bias related to bone quality and to the osteotomy preparation. The degree of underpreparation was standardized on the base of implant dimensions and bone quality. Undersized osteotomies showed a greater remodeling of peri-implant cortical bone during the early healing period compared to nonundersized preparations as demonstrated by Stocchero et al. [30].

Some limits of the present research must be acknowledged: the limited sample size and the primary stability evaluated only on the base of insertion torque values and without resonance frequency analysis can be considered limiting factors [31, 32]. Further research including a greater sample size and a longer follow-up period would be useful to confirm the present outcomes.

## 5. Conclusions

The outcomes of the present research highlighted that dental implants with an increased depth of the threads presented higher insertion torque values without a statistically significant difference compared with standard threads. No differences in bone resorption over time were noted among the two implant morphologies. Deeper threads might be useful in implant sites with low bone quality and in immediate loading rehabilitations when the obtainment of primary stability is a fundamental prerequisite.

## Figures and Tables

**Table 1:** Main demographic data.

	Mean	SD	% (cases/tot)
Age (years)	61.7 (range: 48-72)	8.0	
Gender (M)			57 (8/14)
Loading (DEL)			64 (9/14)
Bone quality			D1: 18 (7/38)
			D2: 40 (15/38)
			D3: 42 (16/38)
			D4: 0
Implants			Syra: 50 (19/38)
			Syra SL: 50 (19/38)

**Table 2:** Correlation between insertion torque and bone quality.

	Final insertion torque (Ncm)			*p*
	Mean	SD	*N*	
Bone quality 1	48.57	3.78	7	*p* = .286
vs. bone quality 2	45.67	8.63	15	
Bone quality 3	45.00	8.76	16	*p* = .833
vs. bone quality 2	45.67	8.63	15	
Bone quality 3	45.00	8.76	16	*p* = .316
vs. bone quality 1	48.57	3.78	7	

**Table 3:** Insertion torque outcomes. The number of exposed threads at 30 and 50 Ncm and the final insertion torque are reported.

	Total		SYRA		SYRA SL	
	Mean	SD	Mean	SD	Mean	SD
30 Ncm (n. visible threads)	1.11	0.66	0.95	0.74	1.26	0.54
50 Ncm (n. visible threads)	0.00	0.00	0.00	0.00	0.00	0.00
Final torque (Ncm)	45.92	7.96	43.42	10.01	48.42	4.10

**Table 4:** Peri-implant health parameters.

Mean (SD)	3 months		6 months		12 months	
	SYRA	SYRA SL	SYRA	SYRA SL	SYRA	SYRA SL
BOP	0.16 (0.38)	0.05 (0.23)	0.32 (0.48)	0.26 (0.45)	0.37 (0.50)	0.58 (0.77)
PD (mm)	1.11 (0.74)	0.95 (0.78)	1.45 (0.57)	1.40 (0.56)	2.01 (0.62)	1.73 (0.79)
PI	0.37 (0.60)	0.32 (0.58)	0.61 (0.64)	0.61 (0.64)	0.74 (0.99)	0.68 (1.00)
Bone resorption (mm)	0.86 (0.78)	0.88 (0.83)	1.17 (0.91)	1.18 (0.92)	1.61 (0.76)	1.72 (1.16)

## Data Availability

Readers can access the data supporting the conclusions of the study by requesting them to the corresponding authors.

## References

[1] Fugazzotto P. A. (2020). Success and failure rates of osseointegrated implants in function in regenerated bone for 72 to 133 months. *The International Journal of Oral & Maxillofacial Implants*.

[2] Szmukler-Moncler S., Salama H., Reingewirtz Y., Dubruille J. H. (1998). Timing of loading and effect of micromotion on bone-dental implant interface: review of experimental literature. *Journal of Biomedical Materials Research*.

[3] Albrektsson T., Brånemark P.-I., Hansson H.-A., Lindström J. (2009). Osseointegrated titanium implants. Requirements for ensuring a long-lasting, direct bone-to-implant anchorage in man. *Acta Orthopaedica Scandinavica*.

[4] Atsumi M., Park S. H., Wang H. L. (2007). Methods used to assess implant stability: current status. *The International Journal of Oral & Maxillofacial Implants*.

[5] Ryu H. S., Namgung C., Lee J. H., Lim Y. J. (2014). The influence of thread geometry on implant osseointegration under immediate loading: a literature review. *The Journal of Advanced Prosthodontics*.

[6] Pesce P., Menini M., Tommasato G., Patini R., Canullo L. (2019). Influence of modified titanium abutment surface on peri-implant soft tissue behaviour: a systematic review of histological findings. *International Journal of Oral Implantology*.

[7] Canullo L., Omori Y., Amari Y., Iannello G., Pesce P. (2018). Five-year cohort prospective study on single implants in the esthetic area restored using one-abutment/one-time prosthetic approach. *Clinical Implant Dentistry and Related Research*.

[8] Canullo L., Pesce P., Tronchi M., Fiorellini J., Amari Y., Penarrocha D. (2018). Marginal soft tissue stability around conical abutments inserted with the one abutment-one time protocol after 5 years of prosthetic loading. *Clinical Implant Dentistry and Related Research*.

[9] Canullo L., Fedele G. R., Iannello G., Jepsen S. (2010). Platform switching and marginal bone-level alterations: the results of a randomized-controlled trial. *Clinical Oral Implants Research*.

[10] Tealdo T., Menini M., Bevilacqua M. (2014). Immediate versus delayed loading of dental implants in edentulous patients’ maxillae: a 6-year prospective study. *The International Journal of Prosthodontics*.

[11] Menini M., Pesce P., Bevilacqua M. (2015). Effect of framework in an implant-supported full-arch fixed prosthesis: 3D finite element analysis. *The International Journal of Prosthodontics*.

[12] Pesce P., Pera F., Bruno D., Menini M. (2018). Survival rate and bone resorption in immediate loading of atrophic maxillary arches using normal and long implants: a pilot observational study. *The International Journal of Prosthodontics*.

[13] Pera P., Menini M., Pesce P., Bevilacqua M., Pera F., Tealdo T. (2019). Immediate versus delayed loading of dental implants supporting fixed full-arch maxillary prostheses: a 10-year follow-up report. *The International Journal of Prosthodontics*.

[14] Abuhussein H., Pagni G., Rebaudi A., Wang H.-L. (2010). The effect of thread pattern upon implant osseointegration. *Clinical Oral Implants Research*.

[15] Lee S. Y., Kim S. J., An H. W. (2015). The effect of the thread depth on the mechanical properties of the dental implant. *The Journal of Advanced Prosthodontics*.

[16] Makary C., Rebaudi A., Sammartino G., Naaman N. (2012). Implant primary stability determined by resonance frequency analysis: correlation with insertion torque, histologic bone volume, and torsional stability at 6 weeks. *Implant Dentistry*.

[17] Misch C. E. (1990). Density of bone: effect on treatment plans, surgical approach, healing, and progressive boen loading. *The International Journal of Oral Implantology*.

[18] Menini M., Setti P., Pera F., Pera P., Pesce P. (2018). Accuracy of multi-unit implant impression: traditional techniques versus a digital procedure. *Clinical Oral Investigations*.

[19] Menini M., Dellepiane E., Pesce P. (2015). Hygienic and dietetic guidelines for implant-supported full-arch immediate loading prostheses. *International Journal of Oral and Dental Health*.

[20] Pesce P., Menini M., Tealdo T., Bevilacqua M., Pera F., Pera P. (2014). Peri-implantitis: a systematic review of recently published papers. *The International Journal of Prosthodontics*.

[21] Pesce P., Canullo L., Grusovin M. G., de Bruyn H., Cosyn J., Pera P. (2015). Systematic review of some prosthetic risk factors for periimplantitis. *The Journal of Prosthetic Dentistry*.

[22] Canullo L., Menini M., Covani U., Pesce P. (2020). Clinical outcomes of using a prosthetic protocol to rehabilitate tissue-level implants with a convergent collar in the esthetic zone: a 3-year prospective study. *The Journal of Prosthetic Dentistry*.

[23] Misch C. E., Strong T., Bidez M. W., Misch C. E. (2008). Scientific rationale for dental implant design. *Contemporary Implant Dentistry*.

[24] Lan T. H., Du J. K., Pan C. Y., Lee H. E., Chung W. H. (2012). Biomechanical analysis of alveolar bone stress around implants with different thread designs and pitches in the mandibular molar area. *Clinical Oral Investigations*.

[25] McCullough J. J., Klokkevold P. R. (2017). The effect of implant macro-thread design on implant stability in the early post-operative period: a randomized, controlled pilot study. *Clinical Oral Implants Research*.

[26] Huang H.-L., Hsu J.-T., Fuh L.-J., Tu M.-G., Ko C.-C., Shen Y.-W. (2008). Bone stress and interfacial sliding analysis of implant designs on an immediately loaded maxillary implant: a non-linear finite element study. *Journal of Dentistry*.

[27] Makary C., Menhall A., Zammarie C. (2019). Primary stability optimization by using fixtures with different thread depth according to bone density: a clinical prospective study on early loaded implants. *Materials*.

[28] Ao J., Li T., Liu Y. (2010). Optimal design of thread height and width on an immediately loaded cylinder implant: a finite element analysis. *Computers in Biology and Medicine*.

[29] Hansson S., Werke M. (2003). The implant thread as a retention element in cortical bone: the effect of thread size and thread profile: a finite element study. *Journal of Biomechanics*.

[30] Stocchero M., Toia M., Jinno Y. (2018). Influence of different drilling preparation on cortical bone: a biomechanical, histological, and micro-CT study on sheep. *Clinical Oral Implants Research*.

[31] Corvino E., Pesce P., Camodeca F., Moses O., Iannello G., Canullo L. (2020). Clinical and radiological outcomes of implants with two different connection configurations, a RCT. *International Journal of Oral Implantology*.

[32] Baldi D., Lombardi T., Colombo J. (2018). Correlation between insertion torque and implant stability quotient in tapered implants with knife-edge thread design. *BioMed Research International*.

